# Seasonality and Children’s Blood Lead Levels: Developing a Predictive Model Using Climatic Variables and Blood Lead Data from Indianapolis, Indiana, Syracuse, New York, and New Orleans, Louisiana (USA)

**DOI:** 10.1289/ehp.7759

**Published:** 2005-02-24

**Authors:** Mark A.S. Laidlaw, Howard W. Mielke, Gabriel M. Filippelli, David L. Johnson, Christopher R. Gonzales

**Affiliations:** ^1^School of Population Health, University of Western Australia, Crawley, Western Australia;; ^2^Department of Basic Pharmaceutical Sciences, College of Pharmacy, Xavier University of Louisiana, New Orleans, Louisiana, USA;; ^3^Department of Chemistry, State University of New York, College of Environmental Science and Forestry, Syracuse, New York, USA

**Keywords:** climate, lead dust, lead exposure seasonality, modeling, PM_10_, soil lead, soil moisture

## Abstract

On a community basis, urban soil contains a potentially large reservoir of accumulated lead. This study was undertaken to explore the temporal relationship between pediatric blood lead (BPb), weather, soil moisture, and dust in Indianapolis, Indiana; Syracuse, New York; and New Orleans, Louisiana. The Indianapolis, Syracuse, and New Orleans pediatric BPb data were obtained from databases of 15,969, 14,467, and 2,295 screenings, respectively, collected between December 1999 and November 2002, January 1994 and March 1998, and January 1998 and May 2003, respectively. These average monthly child BPb levels were regressed against several independent variables: average monthly soil moisture, particulate matter < 10 μm in diameter (PM_10_), wind speed, and temperature. Of temporal variation in urban children’s BPb, 87% in Indianapolis (*R*^2^ = 0.87, *p* = 0.0004), 61% in Syracuse (*R*^2^ = 0.61, *p* = 0.0012), and 59% in New Orleans (*R*^2^ = 0.59, *p* = 0.0000078) are explained by these variables. A conceptual model of urban Pb poisoning is suggested: When temperature is high and evapotranspiration maximized, soil moisture decreases and soil dust is deposited. Under these combined weather conditions, Pb-enriched PM_10_ dust disperses in the urban environment and causes elevated Pb dust loading. Thus, seasonal variation of children’s Pb exposure is probably caused by inhalation and ingestion of Pb brought about by the effect of weather on soils and the resulting fluctuation in Pb loading.

## 

Lead poisoning causes permanent neurologic, developmental, and behavioral disorders, particularly in children. The identification and removal of new sources of human exposure to Pb over the past several decades have significantly reduced the percentage of Pb-poisoned children in the United States, although one remaining population that has not seen this improvement is urban children, particularly from minority groups (Macey et al. 2001) or families with low socioeconomic standing (Mielke et al. 1999). Although some of this continued Pb poisoning is due to remaining point sources (e.g., paint dust from poorly maintained homes), it appears that a significant additional source of Pb contamination is from soil (Filippelli et al. 2005; Mielke et al. 1983)—the legacy of 100 years of Pb use in cities linked to multiple sources (e.g., leaded gasoline, leaded paint, smelters). Recent work has suggested that seasonal increases in children’s blood Pb (BPb) levels relate to exposure via activity, that is, summer days of outdoor play and open windows and doors leading to increased contact with Pb-contaminated soils (Haley and Talbot 2004; Mielke and Reagan 1998; Yiin et al. 2000). Here we suggest an additional possibility—that higher children’s BPb levels may be related to a combination of weather, soil moisture, and wind that effectively remobilizes and makes more bioavailable the diffuse soil Pb. This process may exacerbate this usual summertime behavioral link, with added impacts on urban children’s health.

### Urban Pb.

In the 1970s, the assumed source of soil Pb contamination was Pb-based house paint (Ter Haar and Aronow 1974). An early study of garden soils conducted in metropolitan Baltimore, Maryland, raised questions about that assumption. Soil around Baltimore’s inner-city buildings, predominantly unpainted brick, exhibited the highest amounts of Pb, and soils outside of the inner city, where buildings were commonly constructed with Pb-based paint on wood siding, contained comparatively low amounts of Pb, suggesting that Pb-based house paint could not account for the observed pattern of soil Pb (Mielke et al. 1983). Similarly, the same pattern was also found in Ottawa, Canada (Ericson and Mishra 1990). The quantity and distribution of soil Pb have been studied in numerous places: cities in Minnesota (Mielke et al. 1984/85); New Orleans, Louisiana (Mielke 1994 ); Milwaukee County, Wisconsin (Brinkmann 1994); Washington, DC (Elhelu et al. 1995); Indianapolis, Indiana (Filippelli et al. 2005; Laidlaw 2001); Syracuse, New York (Johnson and Bretsch 2002); Oslo, Norway (Tijhuis et al. 2002); and Ibadan, Nigeria (Sridhar et al. 2000). All these cities exhibited the same distance decay characteristic of high soil Pb contamination in the inner city and decreasing contamination toward the outer parts of the city as initially identified in garden soils of Baltimore (Mielke et al. 1983). Further, similarities in this distance decay pattern of soil Pb supports the idea that Pb-based house paint was not the sole source contributing to these observed differences.

### Sources of Pb.

Except for storage batteries, paint and gasoline additives were the two major high-volume products containing Pb; about the same quantity of Pb, 5 to 6 million metric tons, was used to manufacture each (Mielke and Reagan 1998). Lead-based house paint sales were phased out in 1978 in response to the Lead Paint Poison Prevention Act (Tong 1990). The major processes that now release Pb-based house paint into the soil are deterioration and especially disturbance of old Pb-based paint by power sanding (Mielke et al. 2001).

In the United States, motor vehicles used gasoline containing tetramethyl and tetraethyl Pb additives from the 1920s to 1986. By the 1950s, Pb additives were contained in virtually all grades of gasoline. By 1986, when leaded gasoline was banned, 5 to 6 million metric tons of Pb had been used as a gasoline additive, and about 75% of this Pb was released into the atmosphere (Chaney and Mielke 1986; Mielke and Reagan 1998). Thus, an estimated 4 to 5 million tons of Pb has been deposited into the U.S. environment by way of gasoline-fueled motor vehicles (Mielke 1994). Accumulation of soil Pb created by leaded gasoline is proportional to highway traffic flow (Mielke et al. 1997).

### Pb content and Pb loading of urban soils.

A critical aspect of Pb accumulated in soils is the relationship between Pb content and Pb loading. Studies in Minnesota and Louisiana examined the issue of Pb loading of the soil (Mielke 1993; Mielke et al. 1992). In large cities of Minnesota and Louisiana, the median soil Pb for various site types measured from 6.0 to 32.25 g/m^2^, and the top 0.025 mm contained 6,000–32,250 μg Pb/m^2^ (557–2,996 μg Pb/ft^2^) (Mielke 1993). When one compares this Pb loading rate with the U.S. Department of Housing and Urban Development’s guideline of 40 μg Pb/ft^2^ [U.S. Department of Housing and Urban Development 1999; U.S. Environmental Protection Agency (EPA) 2001] for interior floors, it becomes evident that soil is an enormous reservoir of Pb dust. Because of the low mobility of Pb in soil, all of the Pb that accumulates on the surface layer of the soil is retained within the top 20 cm (Laidlaw 2001; Mielke et al. 1983). The half-life of Pb in surface soils has been estimated to be approximately 700 years, so without corrective action, Pb dust will persist for many generations (Semlali et al. 2004). The persistence of the Pb burden that has accumulated in soil has significant long-term public health implications (Lejano and Ericson 2005).

### Anthropogenic soil Pb speciation and bioavailability.

Pb deposited by human activity onto and retained by surface soils has been added to the relatively small quantities of Pb naturally occurring in the soil. This anthropogenic Pb is generally speciated in the highly bioavailable carbonate, iron, and manganese hydroxide soil fractions, whereas the Pb in natural soils is speciated in the residual, or nonbioavailable fractions (Chlopecka et al. 1996; Lee 1997). Therefore, dust originating from urban soils contaminated by anthropogenic Pb is more toxic than naturally occurring Pb dust. Lead is associated with the smallest particles, the clay grain size fraction in urban soils (Dong et al. 1984); therefore, Pb in dust originating from urban soils is more potent and concentrated than would be expected from simple measurements of the Pb content of the soil (Young et al. 2002).

Bioavailability is indicated by an isotope study of BPb and soil Pb. Each source of Pb has an isotopic signature that is unique to a particular mine. When this characteristic of Pb was first described, most manufacturers began interchanging Pb mining sources, and the effect was to scramble the isotope signatures and render Pb isotopes essentially useless for source identification. The former Soviet Union, however, did not scramble Pb sources that were used in gasoline. Armenia eliminated the use of leaded gasoline before 1997 (Kurkjian and Flegal 2003). The half-life of BPb is about 30 days and is therefore cleared from the blood in a matter of months. If Pb exposure continues, then BPb remains elevated. A study conducted in Yerevan, Armenia, 2 years after the elimination of leaded gasoline indicated that the soil Pb from previous gasoline Pb emissions persisted as a route of exposure for adults (Kurkjian and Flegal 2003). The Pb isotopes of the BPb of adults and the Pb isotopes in contaminated soils were identical, and this provided strong evidence that prior leaded gasoline emissions persist and are highly bioavailable as a route of exposure (Kurkjian and Flegal 2003).

### Seasonal changes in BPb concentration.

Average monthly BPb of children from urban areas tends to increase significantly in summer months (Billick et al. 1979; Blatt and Weinberger 1993; Haley and Talbot 2004; Hayes et al. 1994; Hunter 1977; Hwang and Wang 1990; Johnson and Bretsch 2002; Johnson et al. 1996; Kimbrough et al. 1994; Marrero et al. 1983; Mielke and Reagan 1998; Rabinowitz and Needleman 1982; Rothenberg et al. 1996; Stark et al. 1980; U.S. EPA 1995, 1996; Yiin et al. 2000). Summertime increases of children’s BPb were so prominent over many years in Syracuse, New York, that researchers concluded that the phenomenon was probably caused by the interaction between climate and soils (Johnson and Bretsch 2002; Johnson et al. 1996). The purpose of this study is to test the hypothesis that children’s exposure as measured by BPb is associated with climate and soil factors affecting Pb dust flux in three cities: Indianapolis, Indiana; Syracuse, New York; and New Orleans, Louisiana. Figure 1 presents a map illustrating the locations of the three cities.

## Materials and Methods

This study differs from previous studies because it uses environmental variables as predictors of children’s BPb concentrations, which does not appear to have been attempted before using an ecologic study design. The U.S. EPA studies in Milwaukee (U.S. EPA 1996) and Boston (U.S. EPA 1995) attempted to model BPb using sinusoidal functions; however, it appears that multiple linear regression using climate and soil moisture variables may be more robust due the high percentage of variation explained in the model (up to 87%). The U.S. EPA models did not attempt to use environmental variables to predict BPb concentrations; however, both studies suggested that Pb from the environment might be causing the child BPb seasonality.

This study’s design is described as an analytic time-trend ecologic study (Morgenstern 1998). In ecologic studies, the unit of analysis is the group rather than the individual. The ecologic unit of analysis in this study is the group of children within the city limits of each city who have had their BPb measured. An ecologic design was selected because it is neither practical nor ethical to draw blood from large groups of children on a monthly basis over a long period. One potential limitation of ecologic studies is known as the ecologic fallacy (Morgenstern 1998): the failure of expected ecologic effect estimates to reflect biologic effects at the individual level. However, the biologic plausibility of the associations found at the ecologic level in this study have been found at the individual level in smaller studies (Aschengrau et al. 1994; Lanphear et al. 2003; Maisonet et al. 1997; Mielke and Reagan 1998; Sheldrake and Stifelman 2003). This supports the biologic plausibility of the suggested model. Another facet of this study is that it uses empiric data from three cities that differ in geographic location and climate. Syracuse (latitude 43° N and longitude 76° W) has a cold continental climate; Indianapolis (latitude 40° N and longitude 86° W) is located in the middle continent region; and, New Orleans (30° N latitude and 90° W longitude) has a southerly and warm Gulf Coast climate. The relationship between BPb, weather, and soil moisture is thus studied in geographically and hence climatically diverse locations.

### Data Sources

The independent variables—average monthly soil moisture, particulate matter < 10 μm (PM_10_), wind speed, and temperature—were obtained from state or federal government data sources. Blood Pb databases for each city were obtained from local or state governmental sources as follows.

#### Indianapolis.

In Indianapolis, Indiana, BPb data for 15,944 children were obtained from the Marion County Health Department (personal communication). Nearly 15% of the children listed in the Indianapolis database were < 1 year (*n* = 2,320), 20% were 1–2 years (*n* = 3,202), 13% were 2–3 years (*n* = 2,078), 19% were 3–4 years (*n* = 3,050), 22% were 4–5 years (*n* = 3,476), and 11% were ≥5 years of age (*n* = 1,820). The BPb measurements were collected using the venous method. PM_10_ data were obtained from the Indiana Department of Environmental Management air monitoring station located at 3302 Englist Avenue (personal communication). Soil moisture data were obtained (personal communication) from actual field measurements of the top 6 inches of soil at Illinois Water Survey soil moisture monitoring site number 81 located near Champaign, Illinois, which is approximately 110 miles west of Indianapolis (Hollinger and Isard 1994). Wind speed and temperature data were obtained from the National Oceanic and Atmospheric Administration (NOAA) National Climatic Data Center (NCDC 2004).

#### Syracuse.

In Syracuse, New York, child BPb data were obtained from the Onondaga County Health Department (personal communication). The child BPb screenings for the Syracuse BPb data set were collected from within the city limits of Syracuse. Approximately 90% of child BPb screenings were obtained from passive sources such as county clinics or physicians, and 10% of the screenings were collected from a mobile bus that screened children at locations including day care centers, prekindergarten centers, and Head Start centers. The bus schedule started in May and ended in September, operating full time in June, July, and August. The bus traveled to different locations each summer period. The bus sampling strategy typically targeted areas that had high percentages of BPb concentrations greater than 20 μg/dL. The child BPb screenings were conducted through a combination of capillary and venous methods. The BPb analysis was completed by laboratories certified by the New York State Department of Health. PM_10_ data were obtained from the New York State Department of Environmental Conservation’s Solvay High School air monitoring site located on Gertrude Avenue (personal communication). Soil moisture data were obtained from NOAA (Fan Y, personal communication), and wind speed and temperature data were obtained from the NCDC (2004).

#### New Orleans.

In New Orleans, Louisiana, the child BPb data were obtained from the Louisiana Childhood Lead Poisoning Prevention Program Office of Public Health (personal communication). The screenings for the New Orleans BPb data set were collected from within the city limits. No known geographic or temporal sampling bias was reported. Eighty-four percent of the screenings originated from private providers such as pediatric clinics, physicians, and family practice physicians. Approximately 70% of the children whose BPb was screened were eligible or enrolled in Medicaid. The BPb levels were analyzed primarily by the following laboratories: Labcorp (Burlington, NC), Tamarac (Centennial, CO), Medtox (St. Paul, MN), Quest Diagnostics (Metairie, LA), and ARUP Laboratories (Salt Lake City, UT). The screening procedures were not reported to have changed between January 1998 and May 2003. The data set used in this study was screened for children that had blood drawn using the venous method. The PM_10_ data were obtained from the Louisiana Department of Environmental Quality (personal communication). The soil moisture data were obtained from NOAA (Fan Y, personal communication), and the wind speed and temperature data were obtained from the NCDC (2004).

### Statistical Analysis

We computed the average BPb concentration in each city using the child BPb measurements for each month. The outcome variable, children’s average monthly city BPb concentration for each city, was regressed against the independent variables average monthly soil moisture, PM_10_, wind speed, and temperature; interaction variables; and monthly dummy variables using backward elimination procedures. The independent variables temperature, PM_10_, and soil moisture were computed as the arithmetic mean, whereas the wind speed was computed as the median. Each model’s entry and criteria were 0.10 and 0.15, respectively. Backward variable elimination enters all of the variables in the block in a single step and then removes them one at a time based on removal criteria. Spearman’s rank correlation coefficient was used to assess the association between variables. Statistical analysis was performed using SPSS (version 11.5; SPSS Inc., Chicago, IL).

The Durbin-Watson (DW) and Lagrange multiplier (LM) statistics were calculated to assess the presence of serial autocorrelation. The LM was calculated by regressing the residuals of a model versus the same residuals shifted backward one value relative to the other residuals. The LM statistic is computed by multiplying the *R*^2^ value of this regression by the number of values in the regression. The DW statistic was calculated using SPSS.

## Results

For each city, we calculated Spearman’s rank correlation coefficient matrices for the variables soil moisture, wind speed, PM_10_, and temperature; interaction variables; and monthly dummy variables (M1 to M11). In Indianapolis, Syracuse, and New Orleans, BPb concentration and soil moisture exhibited inverse correlations of –0.41, –0.75, –0.47, respectively. The correlations are presented in Tables 1–3.

### 

#### Regression results: Indianapolis.

The time period of the regression consisted of 36 months between December 1999 and November 2002. The dependent variables for the first model consisted of the average monthly child BPb from a data set of 15,969 children. This model was run using backward elimination procedures.

**Figure 2:**
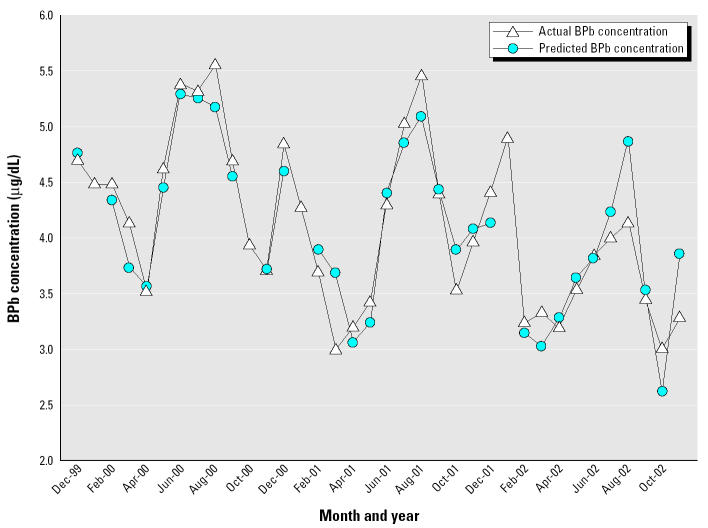
Actual monthly average BPb versus predicted monthly average BPb in Indianapolis, Indiana, for a 36-month period between December 1999 and November 2002 (*n* = 15,969, *R*^2^ = 0.87, *p* = 0.0004, DW = 1.71, LM = 0.85).

This model indicates that the variables or interaction variables including soil moisture, wind speed, PM_10_, temperature, and the monthly dummy variables for March, April, June, July, August, and September explain 87% of the variation in the response variable, monthly average child BPb concentration (*R*^2^ = 0.87, *p* = 0.0004). The DW and LM statistics indicate that the model did not display serial autocorrelation (DW = 1.73, LM = 0.24). Figure 2 presents a chart of the average monthly child BPb concentration for the entire data set versus the predicted child BPb concentration.

The model regression coefficients indicate that the seven predictors with *p*-values less than 0.05 are temperature (*p* = 0.00093), wind speed (*p* = 0.00093), the interaction between temperature and wind (*p* = 0.002), soil moisture (*p* = 0.006), the interaction between soil moisture and temperature (*p* = 0.0076), the interaction between wind and soil moisture (*p* = 0.011), and the interaction between wind and PM_10_ (*p* = 0.016).

#### Regression results: Syracuse.

The time period of the regression consisted of 51 months from January 1994 through March 1998. The use of a mobile clinic to screen children in Syracuse in high-risk areas may have biased the high aggregate monthly average during the months of May through September. However, starting with the 1996 data, the universal screening requirement of the New York State Department of Health (2002) went into effect; subsequently, higher screening rates and more random sampling were apparent in the results. The dependent variables consisted of the average monthly child BPb concentration of a data set of 14,467 children from Syracuse. The model was run using backward elimination procedures. The time-series difference method was used to correct for serial autocorrelation.

**Figure 3:**
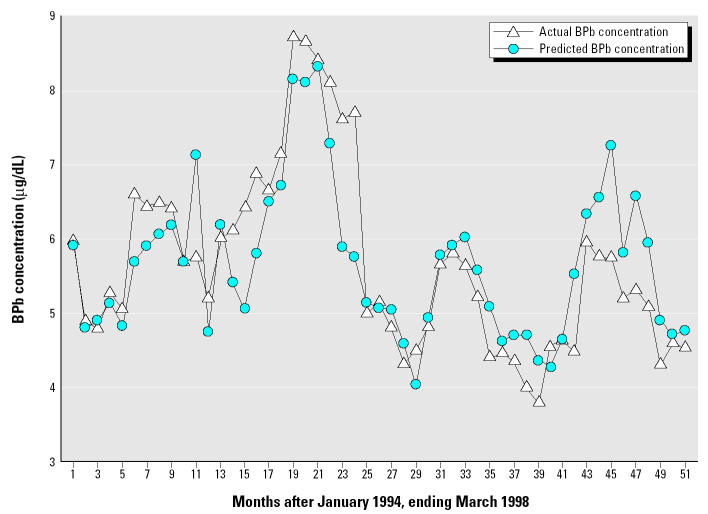
Actual monthly average BPb versus predicted monthly average BPb in Syracuse, New York, for a 51-month period between January 1994 and March 1998 (*n* = 14,467, *R*^2^ = 0.61, *p* = 0.0012, DW = 2.05, LM = 0.049).

This model indicates that the variables or interaction variables including soil moisture, wind speed, PM_10_, temperature, and the monthly dummy variables for January, March, April, May explained 61% of the variation in the response variable, monthly average child BPb concentration (*R*^2^ = 0.61, *p* = 0.0012). The DW and LM statistics indicate that the model did not display serial correlation (DW = 2.05, LM = 0.049). Figure 3 presents a chart of the average monthly child BPb concentration versus the predicted child BPb concentration.

The model regression coefficients indicate that the four predictors with *p*-values less than 0.05 are the interaction between temperature and PM_10_ (*p* = 0.0004), PM_10_ (*p* = 0.0047), wind speed (*p* = 0.029), and the interaction between soil moisture and temperature (*p* = 0.042).

#### Regression results: New Orleans.

The time period of the regression consisted of 65 months from January 1998 through May 2003. The dependent variable is the average monthly blood level of a data set of 2,295 children. This model was run using backward elimination procedures.

**Figure 4:**
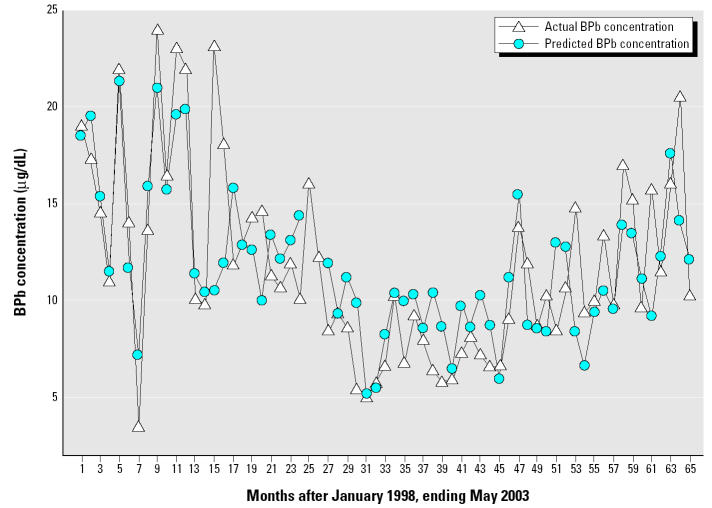
Actual monthly average BPb versus predicted monthly average BPb in New Orleans, Louisiana, for a 65-month period between January 1998 and May 2003 (*n* = 2,295, *R*^2^ = 0.59, *p* = 0.0000078, DW = 1.71, LM = 0.85).

The model indicates that the variables soil moisture, wind speed, PM_10_, temperature, several interaction variables, and the monthly dummy variables for January, February, March, April, July, and October explained 59% of the variation in the response variable, monthly average child BPb concentration (*R*^2^ = 0.59, *p* = 0.0000078). The DW and LM statistics indicated that the model did not display serial autocorrelation (DW = 1.71, LM = 0.85). Figure 4 presents a chart of the average monthly child BPb concentration versus the predicted child BPb concentration.

The model regression coefficients indicate that the predictors with *p*-values < 0.05 are PM_10_ (*p* = 0.00003), the interaction between PM_10_ and wind (*p* = 0.00005), the interaction between PM_10_ and temperature (*p* = 0.0006), and soil moisture (*p* = 0.006). A summary of the statistics from the three cities is presented in Table 4.

## Discussion

### 

#### Soil moisture and soil suspension.

Numerous studies have demonstrated that soil moisture concentration is a significant control of dust (PM_10_) suspension and loading (Chen et al. 1996; Clausitzner and Singer 1996, 2000; Cornelis and Gabriels 2003; Nickovic et al. 2001). Soil moisture is a predictor of wind erosion because soil moisture contributes to bind particles together (McKenna-Neuman and Nickling 1989). Soil particles will become deflated when destabilizing forces such as drag, lift, and aerodynamic forces become greater than stabilizing forces such as particle weight and interparticle binding forces (Iverson et al. 1976).

The threshold shear velocity of a particle is the wind velocity required to deflate (suspend) a particle in the atmosphere (Cornelis and Gabriels 2003). Most models that predict wet threshold shear velocity (*u*_tw_) of a particle take the form


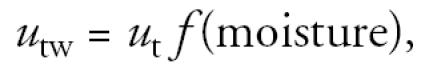


where *u*_t_ is the threshold shear velocity under dry conditions. The function *f* (moisture) is a function of the surface moisture expressed in terms of moisture content *w* (kilogram per kilogram) or capillary potential (Pascal) (Cornelis and Gabriels 2003).

Most models of the threshold shear velocity predict a rise in deflation threshold with increasing moisture content (Cornelis and Gabriels 2003).

With decreasing soil matrix potential from a dry soil, the *u*_tw_ will increase exponentially until a soil matrix potential of –1.5 MPa occurs, at which no soil deflation takes place. The matrix potential (ψ) has been found to be a function of temperature (*T* ), air humidity (*e*/*e*_s_), molar volume of water (*V*_w_; 0.0224 m^3^/mol), and the universal gas constant (*R*; 8.3145 J/mol K) (Edlefson and Anderson 1943):


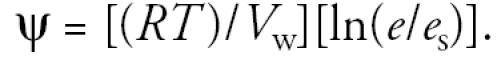


These equations suggest that when temperature is high and soil moisture is low in the summertime, soils are susceptible to deflation. The modeling approach used in this study may have successfully explained the temporal variation in BPb because the matrix potential variables soil moisture (volumetric water content) and temperature were incorporated, which permits prediction of when soils are susceptible to dust emission. The variables PM_10_ and atmospheric Pb represent the end product of dust generation, and the variable wind speed may contribute to the explanation of the variance because of its effect on the PM_10_ (dust) deposition rate. Essentially, the high *R*^2^ values suggest that these variables predict temporal dust generation and exposure of children to Pb from dust in the environment.

The regression models indicate that environmental variables from outside the home, adjusted for seasonality, such as soil moisture, PM_10_, temperature, and wind speed, are significant predictors (*p* < 0.05) of children’s seasonal BPb fluctuations. This suggests that the Pb controlling the seasonal fluctuations originates from the outdoor environment. In the three cities studied here, the urban soils are highly contaminated by Pb (Filippelli et al. 2005; Johnson and Bretsch 2002; Laidlaw 2001; Mielke 1994; Mielke et al. 1999). Thus, there is an abundant source of Pb in urban soils that could be suspended, resulting in elevated Pb dust loading rates during certain weather conditions.

The hypothesis that urban soils are being resuspended into the atmosphere is also supported by the literature that indicates a strong relationship between the suspension of surface soils and atmospheric particulates. In Bakersfield, California, 74% of PM_10_ from July through September 1988 was composed of geologically originated materials (Young et al. 2002). One study estimated that street dust was composed of approximately 76% soil materials (Hopke et al. 1980), and another study estimates that soil contributes between 57 and 90% of road dust (Hunt et al. 1993). Finally, 43% of Pb emissions in the South Coast Air Basin in California resulted from the resuspension of soil and road dust (Lankey et al. 1998).

#### Blood Pb seasonality.

Blood Pb seasonality suggests that Pb exposure varies over time. Thus, those who postulate that Pb-based paint is the primary source of Pb exposure also theorize that dust generation from Pb paint is somehow related to accelerated flaking from painted surfaces during summer months. Some have suggested that the opening and closing of windows painted with Pb paint may produce seasonal exposure to Pb dust (Haley and Talbot 2004). However, this study indicates that soil moisture, PM_10_, wind speed, and temperature fluctuations, adjusted for each other, are very strongly associated with children’s BPb levels. If these variables were noncausally associated with BPb fluctuations, and Pb paint was the source of the seasonality, this would imply that the opening and shutting of doors was associated with soil moisture, PM_10_, wind speed, and temperature. This appears to be counterintuitive because the opening and shutting of windows is likely temperature dependent and not dependent on soil moisture, PM_10_, and median wind speed.

We propose that BPb seasonality results from three or four exposure routes: First, children are likely seasonally exposed to elevated dust Pb loading on interior and exterior surfaces via hand-to-mouth processes, and the elevated Pb loading likely results from seasonal high Pb loading rates due to suspension of urban soils. Second, children are also exposed to direct ingestion of urban Pb-contaminated soil during warmer months. Third, it is possible that children are being seasonally exposed to Pb particles derived from the seasonal opening and shutting of windows painted with Pb paint. Fourth, children are exposed through inhalation to elevated atmospheric dust Pb concentrations resulting from seasonal soil suspension.

In addition, on the basis of the relationship between BPb, high temperatures, low soil moisture, and PM_10_ found in this study, we infer that arid climates with major urban areas and a long-term historical use of Pb in petroleum will experience high sustained rates of Pb loading that originate from Pb dust in soils. We also expect a more prolonged exposure when compared with colder climate areas, resulting in a muting of seasonality trends. These regions may include areas such as Los Angeles, California (USA), Mexico City and Tijuana, Mexico; arid areas of China, Pakistan, and India; and Nigeria, Saudi Arabia, and Cairo, Egypt. Furthermore, the aridity may exacerbate Pb exposure and childhood poisoning, particularly in emerging economies, where leaded gasoline is still in use (Nriagu et al. 1996; Sridhar et al. 2000). Soil Pb may be an important exposure variable in these environments, possibly overwhelming exposure to other sources of Pb.

#### Soil Pb and exposure.

A growing body of research supports the conclusion that urban soils contribute significantly to child BPb poisoning (Mielke and Reagan 1998). Several ecologic studies have found associations between urban soil Pb concentrations and children’s BPb concentrations. A significant logarithmic relationship was reported between soil concentration (> 3,000 sampling points) and child BPb in New Orleans by census tract (*R*^2^ > 0.65) (Mielke et al. 1997). An independent study found a similar relationship in Syracuse (*R*^2^ > 0.65) (Johnson and Bretsch 2002). Both these studies show that, non-temporally, soil accounts for a significant amount of the variation in BPb on a spatial basis. A study of urban dusts and soils in Britain (Culbard et al. 1988) found that soil and outdoor and indoor dusts were the most significant predictor variables in the regression model used to explain children’s BPb levels. The study also found that Pb in interior paint was not a strong independent variable in the final stepwise regression analysis used to explain children’s blood levels (Culbard et al. 1988). A pooled study of 12 epidemiologic studies found that dust Pb loading and soil Pb concentration were the two most significant predictors of children’s BPb levels (Lanphear et al. 1998). In Bunker Hill, Idaho, structural equation modeling indicated that 40–50% of the BPb is from house dust, whereas approximately 30% was from community-wide soils and 30% from the yard at the home and the immediate neighborhood (von Lindern et al. 2003). In Tijuana, Mexico, several studies have found associations between soil Pb and children’s BPb levels (Ericson and Gonzalez 2003; Gonzalez et al. 2002).

The epidemiology literature has also indicated that the removal of Pb-contaminated soil results in significant reductions in child BPb concentration and supports the causal spatial relationship between soil Pb and BPb that has been found in the ecologic studies (Johnson and Bretsch 2002; Mielke et al. 1997, 1999). Soil Pb abatement resulted in a 2.25–2.70 μg/dL reduction in BPb levels when a randomized trial of soil abatement was conducted (Malcoe et al. 2002). Logistic regression indicated that soil Pb > 165 mg/kg was independently associated with BPb concentrations > 10 μg/dL [odds ratio (OR) = 4.1; 95% confidence interval (CI), 1.3–12.4]. Yard soil removal resulted in a 3-fold reduction in the child BPb concentrations of children in the Silver Valley of Idaho, located near the Bunker Hill Superfund site, and reduced the dust Pb levels inside the homes (Sheldrake and Stifelman 2003). Removal of soil from children’s yards reduced the children’s BPb when compared with controls (OR = 0.28; 95% CI, 0.08–0.92) (Maisonet et al. 1997). A study conducted on the effect of soil removal on child BPb concentrations at homes where the soil Pb concentration was greater than 500 mg/kg showed a statistically significant difference between BPb concentrations in homes in which soil was removed versus those where contaminated soil was not removed (*p* < 0.05) (Lanphear et al. 2003).

#### Integrated Exposure Uptake Biokinetic Model for Lead in Children.

The degree to which the proposed exposure hypotheses result in reasonable predictions for BPb levels can be examined with the U.S. EPA’s Integrated Exposure Uptake Biokinetic Model for Lead in Children (IEUBK) (U.S. EPA 1994). To develop an exposure regime for the IEUBK model input, we note that indoor residential dusts generally show Pb concentrations about two times higher, on average, than the corresponding outdoor soils, although these results may have been influenced by fine particulate automotive Pb emissions (Clark et al. 1988; Rabinowitz et al. 1985; Thornton et al. 1990). More recent data are available as summaries from 299 residential locations in eight different Idaho communities for the Human Health Risk Assessment for the Coeur d’Alene Basin showed an average enrichment factor of 1.6 for Pb concentration in carpet dusts compared with outdoor soils (TerraGraphics and URS Greiner 2001; von Lindern et al. 2003). Studies also indicate that dust Pb loading was 1.2 times higher in spring and fall than in winter and that in summer the loading was 1.6 times higher than in winter (Yiin et al. 2000). Model predictions were obtained by specifying seasonal dust concentration differences that would result in the observed dust Pb loading differences. Using the IEUBK default values for Pb in air, water, food, and soil, soil and dust ingestion rates, and with soil representing 45% of the combined soil and dust ingestion, dust Pb concentration was specified as 333 ppm or 550 ppm. For children 1–2 years of age, this increased BPb values from 5.5 μg/dL (winter) to 7.0 μg/dL (summer). When the soil ingestion was specified as 25% of the combined soil/dust intake, the predictions ranged from 5.8 μg/dL (winter) to 7.8 μg/dL (summer).

A more realistic exposure regime for Syracuse might specify a soil Pb concentration of 150 mg/kg, with a winter–summer range of 200–320 mg/kg for the indoor dusts. If the ingested soils are limited to 10% of the total ingestion for combined soils and dusts, the predicted BPb values range from 4.5 to 5.9 μg/dL. This corroborates the range of observations for the 1997–1998 monitoring (Figure 3). Mean observed BPb values for recent Indianapolis data are lower than the 1994–1998 results in Syracuse, so IEUBK model input values would have to be lower to replicate the observations. If one assumes soil Pb is 100 mg/kg and ingestion of soil represents 10% of the total soil/ dust ingestion, and the dust concentration varies from 100 to 180 mg/kg and is associated with a bioavailability of 40% instead of the 30% default value, predicted BPb values range from 3.6 to 4.9 μg/dL. This shows reasonable agreement with the observations (Figure 2).

These exploratory uses of the IEUBK are meant only to indicate the types of concentration values and changes in physiochemical parameters that might provide a mechanistic explanation for the correlations observed in this work. A temporal structure in BPb levels can be modeled by the multicompartmental biokinetic IEUBK model using a wide variety of ingestion rates, soil and dust concentration values, and bioavailability parameters. Because we posit the seasonal variations in PM_10_, it is not unreasonable to consider changes in model default parameters for bioavailability. Small particle size is known to increase Pb uptake from particles (Rieuwerts and Farago 1995; Steele et al. 1990; Wixson and Davies 1994). The potential influence of soil resuspension processes in modulating BPb levels needs careful examination, and future studies should incorporate detailed monitoring for temporal resolution of suspended Pb per volume of air, seasonal influences on residential dust Pb loading and concentration, and measures of Pb bioavailability. Further discussion about the many influences that the natural environment has on public health may be found in Selinus et al. (2005).

## Conclusion

A conceptual model of child BPb seasonal Pb poisoning is suggested. Lead from multiple sources has accumulated in soils of urban environments. The seasonal resuspension of Pb-contaminated soil in urban atmospheres appears to be controlled by soil moisture and climate fluctuations. This study indicates that higher urban atmospheric Pb loading rates are experienced during periods of low soil moisture and within areas of Pb-contaminated surface soils. Children and adults living in urban areas where surface soils are contaminated with Pb may become exposed through indoor and outdoor inhalation of Pb dust and ingestion of Pb deposited within homes and outdoor surfaces. Because resuspension of Pb from contaminated soil appears to be driving seasonal child BPb fluctuations, concomitantly, we suggest that Pb-contaminated soil in and of itself may be the primary driving mechanism of child BPb poisoning in the urban environment.

## Figures and Tables

**Figure 1:**
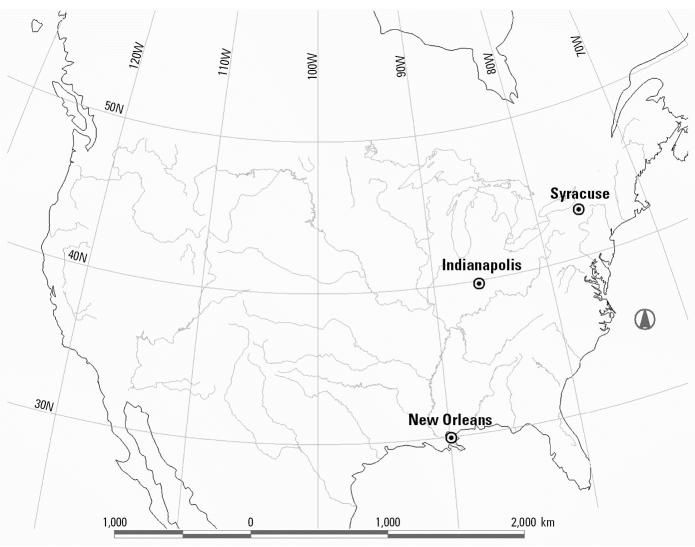
Map showing the locations of Indianapolis, Indiana; Syracuse, New York; and New Orleans, Louisiana.

**Table 1:** Indianapolis: Spearman’s correlation matrix (December 1998 to November 2002).

	SM	W	PM_10_	Temp
BPb all	−0.41	−0.36	0.16	0.20
SM		0.55	−0.13	−0.66
W			−0.18	−0.65
PM_10_				0.19

Abbreviations: SM, soil moisture; Temp, temperature; W, wind speed.

**Table 2:** Syracuse: Spearman’s correlation matrix (January 1994 to March 1998).

	SM	W	PM_10_	Temp
BPb all	−0.75	−0.28	0.10	0.43
SM		0.61	−0.19	−0.57
W			−0.48	−0.66
PM_10_				0.49

Abbreviations: SM, soil moisture; Temp, temperature; W, wind speed.

**Table 3:** New Orleans: Spearman’s correlation matrix (January 1998 to May 2003).

	SM	W	PM_10_	Temp
BPb all	−0.47	0.03	−0.05	−0.16
SM		0.18	−0.12	−0.13
W			−0.24	−0.48
PM_10_				0.59

Abbreviations: SM, soil moisture; Temp, temperature; W, wind speed.

**Table 4:** Multiple linear regression modeling results: all three cities.

City	*R*^2^	df	*F*-value	*p*-Value	SE	DW	LM	Month	No.	Time-series transform
Indianapolis	0.87	16	6.43	0.0004	0.39	1.73	0.24	36	15,969	No transform
Syracuse	0.61	15	3.52	0.0012	0.51	2.05	0.049	51	14,467	Difference
New Orleans	0.59	13	5.33	< 0.00001	3.58	1.71	0.85	65	2,295	No transform
